# Evaluation of Potential Drug-Drug Interactions with Medications Prescribed to Geriatric Patients in a Tertiary Care Hospital

**DOI:** 10.1155/2018/5728957

**Published:** 2018-10-09

**Authors:** Varsha Shetty, Mukta N. Chowta, Nithyananda Chowta K, Ashok Shenoy, Ashwin Kamath, Priyanka Kamath

**Affiliations:** Department of Medicine and Pharmacology, Kasturba Medical College, Mangalore, Manipal Academy of Higher Education, Mangalore, India

## Abstract

**Background and Objectives:**

The drugs most commonly implicated in major potential interactions are those used in the day-to-day clinical management of elderly patients with chronic diseases. This study is planned to evaluate the profile of drug-drug interactions in the medications prescribed to elderly population and also to identify the possible predictors for potential drug-drug interactions in the elderly.

**Methods:**

This cross-sectional study included patients aged above 60 years with a minimum of two drugs in the prescriptions. Data were collected from medical prescriptions and patients' medical records. The data collected included demographic characteristics such as age, gender, height, weight, educational status, socioeconomic status, medical history, and medications prescribed. The prescriptions were analyzed for the potential drug interactions using Lexi-Interact™ Online, an online software to check drug-drug interactions.

**Results:**

A total of 209 patients were included in the study, among them 104 (49.8%) were males and 105 (50.2%) were females. The mean number of medications received was 6.53 ± 2.15 per prescription. Around 138 (66%) patients received more than six medications. The mean number of potential drug interactions seen in the prescription of these patients was 3.17 ± 2.78. Around 18.2% patients had more than five drug interactions. Major drug interactions were observed in 21.42% of cases. Around 3.02% of drug interactions belonged to risk category X, i.e., to be avoided. Logistic regression analysis showed that age above 70 years was associated with the presence of drug interactions. Increased number of medication was independently associated with the occurrence of drug interactions. The presence of drug interactions was not associated with increased number of comorbidities.

**Conclusion:**

A significant number of potential drug-drug interactions were seen in the prescriptions of elderly patients. Increasing age and polypharmacy were identified as the predictors of potential drug interactions.

## 1. Introduction

Alteration in the efficacy or toxicity of one drug due to the presence of another simultaneously administered drug is termed as drug-drug interactions (DDIs). This alteration is mostly quantitative, i.e., the response to a drug is either increased or decreased in intensity. DDIs may occur due to pharmacokinetic processes, i.e., the delivery of a drug to its site of action is altered by a second drug or due to pharmacodynamic processes, i.e., when the two drugs act on same or interrelated target resulting in synergistic or antagonistic activity. Clinically relevant drug-drug interactions may occur with drugs exhibiting steep dose-response curve/narrow therapeutic index, drugs causing microsomal enzyme induction/inhibition, drugs following zero-order elimination kinetics, severely ill patients, in the presence of significant renal/hepatic impairment and elderly patients receiving multiple drugs [1].

Drug therapy is an integral part of patient management. Though the use of multiple drugs may be required either to manage a single disease or comorbidities, harmful interactions may occur between these drugs. The enthusiasm to use new drugs may lead to DDIs that are yet to be identified. Adverse drug reactions may occur as the consequence of DDIs and clinicians may be unaware of the clinical risks of some drug combinations. Drug-drug interactions are a significant cause of hospital admissions and hospital visits, thereby contributing to a huge economic burden. Gathering more and more information on DDIs could help to reduce such adverse effects from DDIs [2–4]. The drugs most commonly implicated in major potential interactions are those used in the day-to-day clinical management of elderly patients with chronic diseases [5]. Few cross-sectional studies involving elderly patients have been conducted to evaluate DDIs, especially in India. Hence, this study is planned to assess the profile of drug-drug interactions in the medications prescribed to elderly population and also to identify the possible predictors for potential drug-drug interactions in the elderly.

## 2. Methods

This cross-sectional study included male and female patients aged above 60 years who attended the Medicine outpatient department of a tertiary care hospital and were prescribed a minimum of two drugs. The study was performed after the approval from the Institutional Ethics Committee, Kasturba Medical College, Mangalore (Ref no. IEC KMC MLR02-16/26). Data were collected for a duration of 6 months, from medical prescriptions and patients' medical records. The data collected included the age of the patient, gender, height, weight, educational status, socioeconomic status, and medical history. Data regarding renal function tests and liver function tests, if available, were recorded. The medications prescribed to the patients were noted and analyzed for potential DDIs.

A sample size of 367 was calculated considering confidence interval of 95% and absolute precision of 5% and the prevalence of DDIs as 45% [6].

The prescriptions were analyzed for the potential drug interactions using Lexi-Interact™ Online, an online software to check drug-drug interactions (http://www.uptodate.com/crlsql/interact/frameset.jsp) [7] available on the website www.uptodate.com. This software provides the severity, risk rating, and the summary of drug-drug interactions.

The risk rating is categorized as A, B, C, D, or X. The progression from A to X is accompanied by increased urgency in the action to be taken. In general, A and B are of academic, but not of clinical concern where as C, D, or X always require attention. Risk category “A” corresponds to no evidence of drug interaction while category “B” denotes the presence of evidence for potential interactions but with little evidence for clinical concern. Hence, for both these categories, no action is required. Monitoring of therapy is recommended for category “C” where there is evidence of potential interaction which is clinically significant. However, the benefits usually outweigh the risks. Dosage adjustments are rarely needed. Therapy modification is to be considered for category “D,” which may involve dose adjustment, considering alternative therapy, aggressive monitoring to minimize toxicity. Drug combinations in the risk category “X” are to be avoided since the risks usually outweigh the benefits. Such drug combinations are considered contraindicated.

DDI can also be categorized by its mechanism, as pharmacokinetic and pharmacodynamic.

### 2.1. Statistical Analysis

For categorical variables, frequencies/percentages were calculated, and continuous variables were expressed as mean ± SD. Comparisons among subgroups were performed using Fisher exact test/Student “*t*” test. The binary logistic regression model was used to analyze the association of occurrence of potential drug-drug interactions with specified risk factors, including gender, age, comorbidities and the number of drugs prescribed. The output of the logistic regression was expressed as adjusted odds ratios with 95% confidence intervals. All tests were performed using a two-tailed test at a significance level of 0.05. SPSS for Windows version 20 (SPSS, Inc., Chicago, IL, USA) was employed for all statistical analyses.

## 3. Results

A total of 209 patients were included in the study. Among them, 104 (49.8%) were males and 105 (50.2%) were females. The mean age of study population was 71.22 ± 7.91 years. The mean number of comorbidities was 2.56 ± 0.94 per patient and the mean number of medications received was 6.53 ± 2.15 per prescription.  Table 1 shows the category-wise number of drug interactions among all patients; 18.2% of patients having more than 5 drug interactions in their prescription. Around 138 (66%) patients received more than six medications. The mean number of potential drug interactions seen in the prescription of these patients was 3.17 ± 2.78 per prescription.

There was a statistically significant difference in the mean age of males and females (males 69.89 ± 7.81 years versus females 72.52 ± 7.83 years, *t* = 2.43, *p*=0.016). There were no significant differences with regard to the number of comorbidities, number of medications, and number of drug interactions between males and females.

The common comorbidities present in the study population were diabetes mellitus (78%), hypertension (73.2%), CVA (26.8%), CAD (10.5%), and dyslipidemia (7.2%). Hypertension was more prevalent in females (*p*=0.013, X^2^ test).

A total of 663 potential drug interactions were seen in 209 patients, with males having 340 and females having 323 encounters. Majority of the drug interactions were pharmacokinetic in nature (Table 2).

Among the total drug interactions, 68.33% belonged to the risk category C, 15.84% were risk category B, and 12.82% were category D. Around 3.02% of drug interactions belonged to risk category X, i.e., to be avoided. There were no differences between males and females with regard to distribution of drug interactions, based on risk category (Table 3).

Table 4 shows the comparison of patients with and without polypharmacy. The mean age between the two groups was comparable. The differences between two groups with regard to the mean number of comorbidity were statistically significant (*p* < 0.002). Patients with polypharmacy (number of medications >6) had a higher number of comorbidity.

There were significant correlations of the frequency of drug interactions with age, the number of medications, and the number of comorbidities (Pearson correlation, *p* < 0.05). Among the comorbidities, hypertension was significantly correlated with the frequency of drug interactions.

Logistic regression analysis showed that age above 70 years is associated with the presence of drug interactions (OR = 1.05; CI: 1.00–1.097; *p*=0.02). Increased number of medication was independently associated with the occurrence of drug interactions (OR = 10.37; CI: 3.35–32.11; *p* < 0.001). The presence of drug interactions was not associated with increased number of comorbidities (Table 5).

Table 6 shows the drug pairs involved in risk X category of drug interactions. The clopidogrel-esomeprazole combination was used in 4 patients.

## 4. Discussion

Elderly patients are the largest consumers of medication. As the elderly patients are more likely to receive multiple medications, potential drug-drug interactions are more likely in this population. Various methods were used to identify and categorize drug-drug interactions. The present study used LexiComp® to evaluate potential drug interactions [7]. Our study showed the mean number of medications per prescription as 6.53 ± 2.15. Earlier studies have also shown the same findings, with a study done at Taiwan [8] showing the mean number medications as 5.8 ± 2.4 and the study done by Teka et al. [9] showing the mean number medication prescribed to elderly patients as 6 ± 4 per patient. Two Indian studies reported a slightly higher values for the mean number of medication received by the patient (7.61 ± 3.37 and 9.15 ± 0.03) [10, 11]. Differences in study settings, number and type of comorbidity as well as the prescribing culture may explain the discrepancy in the findings. In our study, 66% of patients had more than six medications in their prescription. Teka et al. also reported that 42.2% patients had <5 prescribed drugs and 62.2% were exposed to at least one potential DDI [9]. Salwe et al. have reported 52.69% of potential drug interactions with 761 medicines prescribed on admission and 52.91% of drug interactions with 548 medicines prescribed on discharge. Potentially severe drug interactions are 6.98% of total potential drug-drug interactions [11]. Björkman et al. reported potential drug interactions in 46% of elderly patients [12]. Girre et al. studied the potential drug interactions in elderly cancer patients and shown that mean number of medications per patient as 4.7 and identified 45 potential interactions, occurring in 32 patients [13]. In a questionnaire-based study, Loya et al. reported the prevalence of polypharmacy as 72.3% and 46.2% were at risk of having at least one potential drug-drug interaction [14]. Turner et al identified 398 potential drug interactions in 300 patients [15]. Chavda et al. found that 58.27% patients had at least one pDDI and the most common pDDIs were pharmacodynamic in nature and of moderate severity. They observed that the number of pDDI increased with the increase in the age of patients and the number of drugs prescribed [16].

Our study has noted that 83.25% had at least one drug interactions. It is of concern to note that 18.2% patients had more than five drug interactions. The total number of drug interactions identified in the present study is 663, which is sufficiently high to warn us to have a careful monitoring for potential drug interactions in the prescriptions of the elderly. It is gratifying to note that most of these interactions were of moderate intensity. It is unfortunate to observe that a small percentage of patients (3.02%) received drug combinations considered as to be avoided (risk category X).

Hypertension and diabetes were the most common comorbidities. Among the comorbidities, hypertension was more prevalent in females. Obviously, patients with more than five drug interactions had a higher number of comorbidities when compared to patients with less than five drug interactions. Univariate analysis showed significant correlations between the frequency of drug interactions and the age, the number of medications, and number of comorbidities. Among the comorbidities, hypertension was significantly correlated with the frequency of drug interactions. However, the multivariate (logistic regression) analysis showed significant association of occurrence of potential drug interactions only with age and number of medications. Patients with age above 70 years had the higher risk of having drug interactions. Delafuente also reported that the older age and the number of drugs prescribed are more likely to lead to major drug interactions [17]. Similar to our findings, earlier studies also identified polypharmacy as one of the predictors for the occurrence of potential drug interactions [18–22].

The risk of potential drug interaction increases from 39% to 100% when patients are on more than six medications compared to when they are on 2-3 medications [23]. Many elderly patients take one or more medications that are not medically required [21]. However, multiple medications may be needed to cure, to slow the progression of the disease, to prevent its complications and to reduce the symptoms of the disease, thereby improving the quality of life in elderly patients. Hence, balancing the risks and benefits of multiple drug therapies, thereby avoiding harmful drug-drug interactions, in geriatrics is a major challenge for health care providers. Management of drug interactions in the elderly may pose difficulty due to frailty, interindividual variation, and disturbed homeostasis. Better prescribing practices in the elderly can reduce the adverse drug reactions to a great extent [24]. Identifying patient characteristics, as the predictors of potential drug-drug interactions, may help to suggest preventive practices and policies to avoid such interactions.

There were 20 encounters of use of to be avoided drug combinations. The clopidogrel-esomeprazole combination was the most commonly used to be avoided drug combination. Many of the drug interactions can be minimized by using alternative medications or congeners that are not associated with drug interactions. For example, instead of omeprazole, pantoprazole can be used in patients receiving clopidogrel, as pantoprazole do not interfere with the activation of clopidogrel. Balanced use of multiple medications to avoid drug interactions requires the wide knowledge of the pharmacology of drugs.

Apart from prescribed medication, patients may also consume nonprescription medications, further adding to the risk of drug interactions. Age-related alterations in pharmacokinetics also pose them to increased risk of clinically significant drug interactions. The quality of life in elderly may be affected by the presence of drug interactions and morbidity may be increased by clinically unrecognized drug interactions. Hence, careful use of medications and strict monitoring is required to avoid drug-drug interactions. Medications given to treat most common comorbidities like diabetes and hypertension should be screened for potential drug interactions. Patient education and counseling by clinicians are essential to avoid improper use of nonprescription drugs.

Limitation of our study needs to be mentioned. This study is a medical record-based study wherein patient's data were collected from the patient's files without direct interaction with the patients and drug interactions identified were potential or theoretical. Studies based on clinical evaluation of the patients for the possibility of drug interactions may be more valid as it provides real-time data. This study is done at a tertiary care hospital, where the prescription pattern may be different, compared with primary care settings. Many elderly patients are treated in primary care settings and hence generalizations of our findings may not be appropriate. Another limitation is that, being a record-based study, nonprescription drugs were not taken into account. However, this study being the first in our settings provides the baseline data that can be used in finding the prevalence of potential drug-drug interactions in elderly patients.

## 5. Conclusion

A significant number of prescriptions of elderly patients had more than five potential drug-drug interactions. A small percentage of drug interactions were belonging to the risk category X (i.e., to be avoided drug combination), which could be easily avoided by using alternative medications or congeners that are not associated with drug interactions. Increasing age and polypharmacy were identified as the predictors of potential drug interactions.

## Figures and Tables

**Table 1:** Number of drug interactions among all patients.

Number of drug interactions	All patients, *n* (%)
0	35 (16.7)
1-2	71 (34)
3–5	65 (31.1)
>5	38 (18.2)

**Table 2:** Distribution of drug interactions based on mechanism (pharmacokinetic and pharmacodynamic interactions).

D/Is	All patients	Male	Female	*p* value
Overall	663	340	323	0.24
Pharmacokinetic, *n* (%)	241(36.35)	117(34.41)	124(38.39)	0.055
Pharmacodynamic, *n* (%)	422(63.65)	223(65.59)	199(61.61)	0.296

X^2^ test.

**Table 3:** Distribution of drug interactions based on risk category.

Risk category of D/Is	All patients	Males	Females	*p* value
B, *n* (%)	105 (15.84)	47 (13.82)	58 (17.96)	0.14
C, *n* (%)	453 (68.33)	247 (72.65)	206 (63.78)	0.53
D, *n* (%)	85 (12.82)	34 (10)	51 (15.79)	0.26
X, *n* (%)	20 (3.02)	12 (3.53)	8 (2.48)	0.28

X^2^ test.

**Table 4:** Comparison of comorbidities between patients with and without polypharmacy.

Variables	Number of medications >6	Number of medications <6	*t* value	*p* value
Age (years)	71.36 ± 7.36	70.93 ± 8.93	0.35	0.73
Number of comorbidities	2.7 ± 0.97	2.28 ± 0.83	3.12	0.002^*∗*^

Values are expressed as mean ± SD. Student “*t*” test. ^*∗*^Significant.

**Table 5:** Predictors of D/Is (multivariate logistic regression analysis).

Variables	Groups	Patients with D/Is	Patients without D/Is	Wald	Odds ratio (95% CI)	*p* value
Age	<70	75	22	4.04	1.05 (1.00–1.097)	0.04
	>70	99	13			

Gender	Male	89	15	0.18	0.86(0.42–1.75)	0.67
	Female	85	20			

Number of comorbidities	1	22	7	0.31	0.87 (0.54–1.42)	0.58
	2	52	15			
	3	75	10			
	4	21	2			
	>4	4	1			

Type of comorbidity	Diabetes	136	27	0.20	0.81 (0.31–2.11)	0.66
	Hypertension	130	23	1.71	1.92 (0.72–5.09)	0.19
	CAD	21	1	0.60	1.56 (0.51–4.82)	0.44
	Dyslipidemia	14	1	0.05	1.17 (0.31–4.46)	0.82
	CVA	51	7	0.38	0.76 (0.32–1.81)	0.54

Number of medications	<6	48	23	16.47	10.37 (3.35–32.11)	<0.001
	>6	126	12			

**Table 6:** Medication pairs involved in risk X category drug interactions (to be avoided).

Medication pairs	Number of D/Is	Effect of drug interaction
Azithromycin-silodosin	1	Increased serum concentration of silodosin
Domperidone-escitalopram	2	Enhanced QTc-prolonging effect
Domperidone-quetiapine	2	Enhanced QTc-prolonging effect
Escitalopram-quetiapine	1	Enhanced QTc-prolonging effect
Atorvastatin-silodosin	2	Increased serum concentration of silodosin
Clopidogrel-esomeprazole	4	Diminished antiplatelet effect of clopidogrel
Ciprofloxacin-domperidone	1	Enhanced QTc-prolonging effect
Domperidone-fluconazole	1	Increased serum concentration of domperidone
Nifedipine-phenytoin	1	Increased serum concentration of phenytoin
Prazosin-tamsulosin	1	Enhanced antihypertensive effect
Domperidone-granisetron	1	Enhanced QTc-prolonging effect
Escitalopram-flupentixol	1	Enhanced QTc-prolonging effect
Bambuterol-salmeterol	1	Enhanced adverse/toxic effects
Amitriptyline-salbutamol + ipratropium	1	Enhanced anticholinergic effect

## Data Availability

The data used to support the findings of this study are available from the corresponding author upon request.
